# Phage Therapy for Bone and Joint Infections: Towards Clinical Translation

**DOI:** 10.3390/antibiotics14121187

**Published:** 2025-11-21

**Authors:** Concha Ortiz-Cartagena, Lucia Blasco, Inés Bleriot, Jaime Esteban, María Dolores del Toro, José Luis del Pozo, María Tomás

**Affiliations:** 1 Multidisciplinary and Translational Microbiology Group (MicroTM), Biomedical Research Institute of A Coruña (INIBIC), Microbiology Service, University Hospital of A Coruña (CHUAC), University of A Coruña (UDC), 15006 A Coruña, Spainluciablasco@gmail.com (L.B.); bleriot.ines@gmail.com (I.B.); 2 MePRAM, Proyecto de Medicina de Precisión Contra las Resistencias Antimicrobianas, 28029 Madrid, Spain; jesteban@fjd.es; 3 CIBERINFEC-CIBER de Enfermedades Infecciosas, 28029 Madrid, Spain; 4 Department of Clinical Microbiology, IIS-Fundación Jiménez Díaz, Universidad Autónoma de Madrid (UAM), 28040 Madrid, Spain; 5 Division of Infectious Diseases and Microbiology, University Hospital Virgen Macarena, 41009 Seville, Spain; 6 Department of Medicine, Biomedicine Institute of Sevilla, University of Sevilla, 41009 Seville, Spain; 7 Department of Clinical Microbiology, Clínica Universidad de Navarra, 31008 Pamplona, Spain; jdelpozo@unav.es; 8 Infectious Diseases Division, Clínica Universidad de Navarra, 31008 Pamplona, Spain; 9 Instituto de Investigación Sanitaria de Navarra (IdiSNA), 31008 Pamplona, Spain

**Keywords:** phage therapy, osteoarticular infections, administration routes, delivery systems

## Abstract

Osteoarticular infections (OAIs), including osteomyelitis, septic arthritis, prosthetic joint infections, and facture-related infections, remain a major challenge due to biofilm formation and the prevalence of multidrug-resistant (MDR) pathogens. Although OAIs are predominantly caused by *Staphylococcus aureus* and coagulase-negative staphylococci, the increasing incidence of MDR Gram-negative infections adds further complexity to their management. Standard approaches, combining surgery and prolonged antibiotic therapy, frequently result in recurrence and poor outcomes. Bacteriophage (phage) therapy has emerged as a promising adjunct or alternative approach, offering high host specificity, replication at the infection site, and activity against biofilm-embedded bacteria. This review highlights recent advances in phage therapy for OAIs, focusing on administration routes (intravenous, intra-articular, topical, and oral) and on novel pharmaceutical delivery systems such as hydrogels, bone cements, microparticles, nanoparticles, and implant coatings. Preclinical and early clinical studies have analyzed phage stability, controlled release, and the synergistic effects of combined phage/antibiotic therapy. However, challenges remain regarding standardization, immunogenicity, and regulatory approval. Nonetheless, phage therapy shows promise for clinical translation as an adjunct or alternative to conventional treatments for OAIs. Well-designed clinical trials are urgently needed to confirm the efficacy of phage therapy, optimize delivery strategies, and integrate the treatments in routine practice. Despite encouraging outcomes for a successful clinical implementation, regulation and standardization of GMP production are required.

## 1. Introduction

Osteoarticular infections (OAIs) comprise a group of conditions that can be classified in two groups: osteomyelitis (involving bone) and septic arthritis (involving joints). The global prevalence of OAIs, especially implant-associated infections and those caused by multidrug-resistant microorganisms, such as Gram-negative bacteria, has increased in recent years. This high prevalence of OAIs is associated with the increased use of medical devices such as joint prostheses [1], mainly because of the higher incidence of conditions such as osteoarthritis in the aging population. Current estimates suggest an incidence of approximately 70 cases of OAIs per 100,000 inhabitants per year in developed countries, with the recurrence rate ranging from 10 to 30%, owing to the increasing number of implant-associated infections. This situation entails a high healthcare cost as well as a decrease in quality of life for the patients involved. In addition, the formation of bacterial biofilms, which significantly reduces the effectiveness of available antibiotics, is a key step in the pathogenesis of OAIs [1,2].

The most common pathogens that cause OAIs are *Staphylococcus* spp., *Streptococcus* spp., and Gram-negative aerobic bacilli (such as Enterobacterales and *Pseudomonas aeruginosa*). Benito et al., 2016 [3] demonstrated that coagulase-negative *Staphylococcus* (CoNS), such as *Staphylococcus epidermidis*, are the most prevalent among these pathogens in chronic infections. In a later study, the same authors demonstrated that *Staphylococcus aureus* is more prevalent in acute infections [4] and that both pathogens together are responsible for up to two-thirds of all such infections [1,2]. OAIs involving *Staphylococcus* are particularly challenging to treat as indicated by the increasing number of reports of infections caused by methicillin-resistant *Staphylococcus aureus* (MRSA) in healthcare-associated infections as well as in community-acquired infections. Moreover, CoNS strains are usually multidrug-resistant, with a high rate of transfer of resistance genes to other *S. aureus* strains, raising serious concern [1].

The above factors reduce the efficacy of the current OAI treatment, which typically consists of long-term combined antibiotic therapy and repeated surgical interventions, often leading to prolonged hospitalization, increased costs, and higher patient mortality [1,2,5]. Developing new therapeutic strategies is therefore of urgent priority to prevent clinical complications, improve patient prognosis, as well as reduce the spread of antibiotic-resistant pathogens in the population [2].

Among the innovative strategies available, bacteriophage (phage) therapy is particularly promising, especially in the context of OAIs, in which the aforementioned pathogens pose a significant challenge [2]. Phage therapy, defined as the use of phages or their derivatives to treat infections caused by antibiotic-resistant bacteria, has several advantages over antibiotics, including the following: (i) high specificity toward the host (at the species and even strain level), which minimizes the impact on the patient’s microbiota and eukaryotic cells; (ii) low doses required, as phages replicate at the infection site; which (iii) significantly enhances therapeutic efficacy; (iv) effectiveness against biofilm and bacteria with low metabolic activity, such as persistent strains. Multiple studies have analyzed the clinical challenges of phage therapy in OAIs.

Although phage therapy is suitable for treating infections, specifically OAIs, the combined use of phages and antibiotics yields a synergistic effect that enhances the action of the treatment relative to each individual therapy [6]. The therapeutic success of PAS is influenced by administration doses and timing. Administration of sublethal levels of antibiotics in combination with phages has been reported to produce the best results. Given that the antibiotic doses alone induce resistance, it seems that the phage activity can prevent the emergence of resistant mutations and in other cases block the efflux pumps that act as phage receptors and restore the antibiotic sensitivity. The dose of phage used depends on the phage itself and is determined by its burst size, adsorption rate and latent period [6,7,8]. The timing and frequency of administration are also important, with the best results being produced by sequential administration of phages followed by antibiotics [6]. PAS therapy has been widely used in the treatment of OAIs and specifically in biofilm implants, taking advantage of the capacity of phages to degrade biofilms, which can facilitate antibiotic access to the bacterial targets [9].

Phage therapy holds great promise for treating OAIs, with treatment efficacy reaching as high as 95.5% when the phage therapy is combined with antibiotics in patients with prosthetic joint infection, as shown by Fedorov et al. (2023) [10]. However, challenges related to standardization, immunogenicity, and regulatory acceptance must be addressed. Improvements in administration routes (e.g., intravenous [IV], injectable, intraoperative topical) and in the formulation used (e.g., hydrogels, micro/nanoparticles, microcapsules, nanofibers, implant coatings, lyophilization) can enhance the phage stability, control release of phage at the infection site, and ensure elimination to prevent adverse effects [11,12,13].

**Figure 1:**
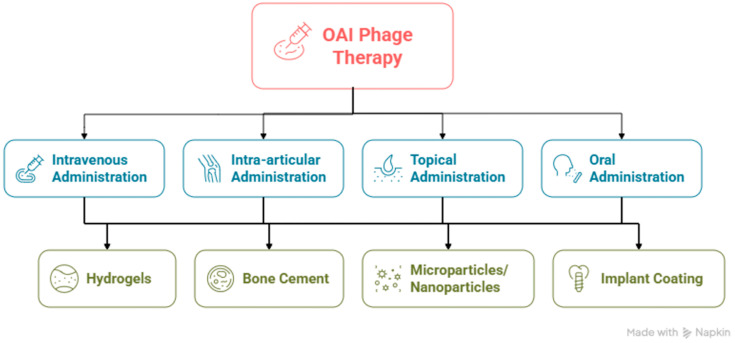
Scheme showing the administration routes and delivery systems used in phage therapy to treat osteoarticular infections (generated by Napkin AI, https://www.napkin.ai/, accessed on 17 November 2025).

In this article, we reviewed articles that have explored several different ways of applying phage therapy in OAIs (including both in in vivo and in vitro studies) and involve PK/PD information (Figure 1), delivery systems for phage therapy in osteoarticular infections, as well regulatory guidelines.

This literature review was conducted by searching for scientific articles and regulatory information on OAIs across several databases, including PubMed (https://pubmed.ncbi.nlm.nih.gov), Google Scholar (https://scholar.google.es), the European Medicines Agency (EMA) (https://www.ema.europa.eu/en/homepage), and the U.S. Food & Drug Administration (FDA) (https://www.fda.gov). The following search terms were used: “*Osteoarticular infection*”, “*OAIs*”, “*OAIs and phage therapy*”, “*phage therapy*”, “*administration routes*”, “*delivery systems*”, “*regulatory*”, “*pharmacokinetics/pharmacodynamics*”, and “*PK/PD*”. The scientific articles were published between 2018 and 2025, except for four studies, published in 2004, 2012, 2016, and 2017, respectively.

## 2. OAI Phage Therapy Pharmacokinetics (PK): Administration Routes

Five main routes of administration of phages have been described [14]. However, in OAIs, the most relevant are intravenous, topical (including intra-articular), and oral. These routes are often combined to improve the therapeutic success with antibiotics.

### 2.1. Intravenous Administration

Intravenous phage therapy is emerging as an option in cases of systemic or severe infections such as OAIs. This strategy involves the direct administration of a phage or a phage cocktail into the bloodstream. Safe administration is ensured by sterile practice and the removal of endotoxins, residual bacterial debris, and solvents. Although treatments administered by this route are generally well tolerated, mild side effects have been reported [15].

### 2.2. Topical and Intra-Articular Administration

Topical phage delivery is usually intraoperative or intra-articular (directly into the joint). Phages can be applied directly, used as an antimicrobial coating on prosthetic material or injected post-surgery via drains for sustained release. Although biofilm formation hinders antibiotics from reaching the bacterial cells, phages can penetrate and degrade biofilms both by lytic activity and depolymerase enzymes [16]. Phages are often combined with antibiotics to enhance efficacy, and proximity to the pathogen at the site of infection is crucial for success of the treatment [15,17].

### 2.3. Oral Administration

The oral route is minimally invasive, cost-effective, and painless; however, phages are often inactivated in the acidic gastric environment. Protection strategies such as encapsulation, freeze-drying, and spray-drying are required to preserve viability and therapeutic activity [18]. While effective in gastrointestinal infections, the use of this route for treatment of OAIs remains experimental.

**Figure 2:**
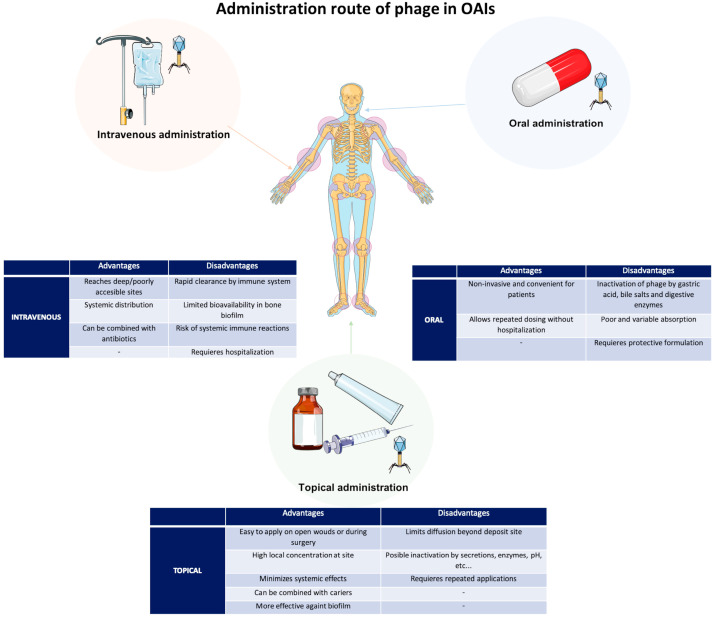
Phage therapy for OAI administration routes. Advantages and disadvantages of the different administration routes.

The choice of the administration route in OIA is conditioned by the infection site, the severity of infection, and patient-specific factors (Figure 2). The topical route, i.e., intra-articular, intra-operational, or local drainage, is commonly used to treat OIAs, either alone or in combination with other routes of administration [17,19]. The topical administration presents several advantages for its use in OAIs, including the high concentration of phages at the delivery site, low immunogenicity, and high efficiency against biofilms. Although the intravenous administration route can induce a good systemic response by developing a strong immune response, it is the second choice of route and is often used in combination with the topical route. The intravenous route is applied in systemic infections or multifocal infections, and it is also used in the absence of surgery or to maintain continuous phage perfusion [20,21]. Finally, oral administration is the least commonly used because of possible inactivation of the phage as well as the protective systems that are required [18,22].

## 3. OAI Phage Therapy Pharmacodynamics (PD)

Several studies have analyzed various aspects of the pharmacodynamics of phage therapy [23,24,25], showing the following: (i) Effective MOI: initial dosing must compensate for loss due to inactivation/biofilm adsorption. Several researchers recommend the use of 10^8^–10^10^ PFU per IA instillation, adapted to joint volume. (ii) Self-amplification: phages replicate if bacterial density is sufficient; requires appropriate host specificity and combination of ≥2 phages to limit resistance. (iii) Phage–antibiotic synergy (PAS): demonstrated with rifampicin, daptomycin, β-lactams. Timing of antibiotic administration is critical to avoid bacteriostatic suppression of bacterial replication during the first replication cycles of phages.

For all of these reasons, we propose the following practical treatment framework:

1. Microbiology: Pathogen isolation; phage screening (efficiency of plating, biofilm activity, stability in synovial fluid and with antibiotics) [26,27,28].
2. Surgery: Debridement, Antibiotics and Implant Retention (DAIR) or revision as indicated; lavage to reduce bacterial load and neutralizing proteins [29,30,31].
3. Administration routes:
  - IA during surgery and via post-op catheter (daily or every 24–48 h for 3–14 days) [32].
  - Local instillation (bone cavity, spacers) [33].
  - IV only as adjunct when local not feasible [20].
4. Antibiotics: Maintain standard biofilm-active regimens (e.g., rifampicin combos for staphylococci, fluoroquinolones for Gram-negatives), with mindful scheduling to enhance PAS [34,35].
5. Monitoring:
  - Synovial fluid aspirates: PFU counts [36], qPCR for phage and bacterial DNA [37].
  - Serum: phage titers [36], anti-phage antibodies if prolonged therapy [38].
  - Clinical/biological markers: pain, drainage, CRP/ESR [39].
6. Safety: Generally well tolerated; adverse effects mild and transient (e.g., fever, chemical synovitis) [11,40,41].

## 4. OAI Phage Therapy Delivery Systems (Pharmaceutical Developments)

A good phage product for therapy must overcome some issues derived from the phage and from the environment. Phages are highly stable in buffer and can be stored for years. However, when used in therapy, factors such as acidity and temperature can affect their stability, reducing the phage titer or leading to loss of infectivity. To counteract the environmental effects on phage stability, several delivery strategies have been developed. These pharmaceutical strategies consist of the modifying the medium viscosity to maintain the phage morphology, modifying the osmotic pressure, and using freeze-drying or spray-drying to dry the phage preparation [15].

In the case of OAIs, IV administration of phages results in a short residence time, and the phage preparations are therefore usually administered in combination with local delivery, which also favors self-replication at the infection site [15]. In these types of infection, optimal administration implies the use of pharmaceutical formulations that increase the contact between the phages and the bacteria adhered to the bone or the prostheses. Novel pharmaceutical formulations for phage delivery in OAI include hydrogels, bone cements, micro/nanoparticles, and implant coatings (Table 1).

### 4.1. Hydrogels

Hydrogels are three-dimensional networks of polymers that can swell in water and retain a large volume of water while maintaining their structure [51,52]. Among the new alternatives available for phage delivery, this strategy has several advantages, including high water absorption, biodegradability, biocompatibility, and efficient material delivery. Moreover, the use of hydrogels ensures heightened phage activity, concentration, controlled release, and strong antibacterial properties. Hydrogels are therefore extensively applied in the biomedical field as wound dressings, oral gels, implantable devices, hydrogel microneedles, and injectable hydrogels [51].

The structures can be physical (ionic, alginate), freeze–thaw (polyvinyl alcohol (PVA), thermal (P407), and chemically cross-linked. Unless physically cross-linked, chemical unions require the addition of chemical agents for the formation of covalent joints, which lead to a sustained release of the phage. However, these agents are often toxic, and the cross-linking process is also more complex [51].

Barros et al. (2020) developed an alginate hydrogel with nanoparticles of hydroxyapatite and loaded this with the phage LM99 of *Enterococcus faecium* [42]. Characterization of this hydrogel showed the following: (i) the phage was contained in the alginate matrix with a load efficiency of 91%; (ii) phage release was pH dependent, with phage delivery in a broad range of pH 5–9, which was compatible with the site of bone infection (pH 6.8); (iii) phage viability was maintained for 60 days in solution, and for 7 days when the alg-nanoHA solution was ionically cross-linked with calcium chloride; (iv) HA has a role in the regeneration of the bone tissue. The study also demonstrated an effective antimicrobial effect of the hydrogel with the LM99 phage in vitro and in vivo assays, using a multidrug-resistant *E. faecalis* strain [42]. In 2020, Wroe et al. [43] developed a hydrogel with adhesive peptides (GRDGSPC or GGYGGGPC(GPP)5 GFOGER(GPP)5GPC) for use in encapsulating *P. aeruginosa* phages. In vitro studies showed a reduction in biofilm formation when use of the hydrogel was compared with that of free phages, and testing of the hydrogel in a mouse model of bone infection showed better recovery from infection with the encapsulated phage treatment.

Clinical studies have also used phages encapsulated in hydrogels. Ferry et al. (2020) used a phage entrapped within the DAC^®^ hydrogel, a hyaluronic acid and polylactic acid hydrogel, to treat a patient with a *S. aureus* knee megaprosthesis infection, observing rapid release of the phages from the hydrogel matrix and its deposition in the prosthesis and titer stability for 6 h [31].

### 4.2. Bone Cement

Polymethylmethacrylate (PMMA) is a polymer commonly known as bone cement. This material is frequently used for implant fixation in orthopedic and trauma surgery. PMMA acts by creating a space that holds the implant against the bone, acting as a space filler [13]. This spacer has long been used as an antibiotic carrier and to a lesser extent as a phage carrier [53]. Loading of the cement with phage can be a good strategy to locally treat OAIs, although some issues must be overcome, such as the maintenance of phage activity over time, in order to treat persistent infections (e.g., biofilms) in prostheses [54].

Samokhin et al. (2018) conducted an experimental study in which they impregnated a PMMA bone cement with *P. aeruginosa* phages pH 20 and pH 57 [44]. The titer stability was one week for the former phage and two weeks for the latter phage [44]. Based on the findings of the aforementioned study, Fedorov et al. (2023) treated a group of 23 patients with periprosthetic joint infections (PJIs) with a combination of selected phages and antibiotics [10]. Before placement of the endoprosthesis components, the bone cement was mixed with a solution of selected staphylococcal phages. Finally, after the surgery, a solution of active staphylococcal phages was injected into the periprosthetic area by drainage in the postoperative wound (or by injection in case or drainage removal). In comparison with a historical cohort, a significantly reduced rate of PJI relapse (4.5% vs. 36.4%) and a higher treatment response rate (95.5% vs. 63.6%) was observed at 12 months.

### 4.3. Microparticles/Nanoparticles

Other delivery systems involve combining phages with delivery systems such as microcapsules, which may enhance the treatment efficacy [13]. A drug or a biological agent such as phage can be microencapsulated by packaging it into several substances like alginate, chitosan, silk fibroin, PLGA (polylactic-co-glycolic), and/or others, to achieve controlled release and stability [55,56,57]. Microparticles can also be used in periprosthetic traumatological infections, in which they can be applied directly by intra-articular administration or incorporated into bone cement [58]. The antimicrobial efficiency of these preparations has been demonstrated in several studies in which the microparticles were loaded with antibiotics [59,60,61,62], but fewer studies have been performed with phage-loaded microparticles [13,57,63,64].

Xu et al. (2024) developed an injectable formulation based on microparticles loaded with a phage against MRSA [13]. This formulation, a compound consisting of silk fibroin microparticles with polyethylamine and phage (Ph-MPs), enables rapid release of the viruses in the first 30 min, followed by a slow and sustained release. An in vivo assay using a murine model showed an antibacterial activity as good as that reached by the treatment with the free bacteriophage and the treatment with the antibiotic vancomycin, respectively. This alternative has several advantages over the use of free phages, as loading phages in microcapsules improves the storage and stability of bacteriophages and also reduces inflammation (lower white blood cell count).

Treating biofilms produced by MDRA pathogens is complicated by the fact that the biofilms are difficult to penetrate, which hampers delivery of the antibiotics and nanoparticles. To solve this problem, Wang et al. (2024) developed a phage–liposome nanoconjugate [45]. These researchers used liposomes loaded with the Sb-1 phage, which degrades the exopolysaccharide matrix of the biofilm. The nanoconjugates efficiently removed a MRSA biofilm, and when tested in the PJI rat model, a decrease in the infection was observed (with a reduction in the bacterial load of more than 1000-fold) and significant promotion of the recovery from osteomyelitis occurred.

### 4.4. Implant Coatings

To prevent colonization and subsequent infection in orthopedic implants, the materials can be treated with phages before surgical implantation. Various coating strategies have been investigated, including the following: (i) stainless steel orthopedic K-wires with a hydroxypropyl methylcellulose matrix incorporating phages; (ii) biopolymer matrices embedding phages; (iii) phage incorporation to hydroxyapatite and beta-tricalcium phosphate (β-TCP); (iv) proteolytic multilayer coating with surface-bound phages.

Meurice et al. (2012) evaluated materials commonly used in bone repair, such as hydroxyapatite and β-TCP phages targeting the *Escherichia coli* K12 strain [46]. The study demonstrated that phages remained viable for at least six days within dense microporous samples, suggesting that phage-loaded ceramics could be used in a prophylactic strategy to support bone regeneration while simultaneously preventing infections. Building on this concept, Kaur et al. (2014) developed stainless steel orthopedic K-wires coated with a hydroxypropyl methylcellulose containing phages, linezolid, or a phage–linezolid combination [47]. The coatings strongly inhibited MRSA adhesion, with the combined formulation showing the most pronounced effect and no emergence of resistant mutants. In a subsequent study, the same group embedded *S. aureus* phages and linezolid within a biopolymer, demonstrating a substantial reduction in bacterial adhesion as well as diminished joint inflammation and faster recovery of locomotor function in a murine implant model, again without selection of resistant strains [50].

Alternative approaches have explored the use of hydrogels as implant coatings. Ismail et al. (2020) embedded *E. coli* λvir phages into a calcium phosphate-based prosthetic material coated with calcium alginate hydrogel, thereby enhancing phage retention and antimicrobial activity [48]. Similarly, 3D-printed calcium phosphate ceramics loaded with a phage cocktail against heterogeneous *S. aureus* and *E. coli* populations successfully prevented biofilm formation on the ceramic surface and reduced osteoblast infection [65].

More recently, Müller et al. (2021) proposed an innovative proteolytic coating strategy that incorporates phages within multilayers of polyelectrolytes (PEMs) using the Layer-by-Layer (LbL) deposition technique [49]. In this system, alternating adsorption of polycations and polyanions creates multilayers capable of retaining phages, particularly in the outermost layers, enabling their gradual diffusion. The study demonstrated that T4 phages and phages targeting *S. aureus* remained viable and active within PEMs composed of polyethyleneimine (PEI) and polyacrylic acid (PAA), highlighting the potential of this approach for biomedical applications. This procedure combines the antibacterial activity of phages with a surface pre-modification using multilayers of polyelectrolytes (PEMs), deposited using the LbL technique. The technique is based on the consecutive adsorption of selected polycations and polyanions from a solution, usually starting with the anchoring of the polycation to the chosen material, followed by the adsorption of the polyanion, and repeating this process for the desired number of cycles. In this approach, the phages are mainly integrated into the outermost layer of the system, allowing them to diffuse into the PEM. In the study, T4 phages or phages against *S. aureus* specifically bind to pre-dosed PEMs of PEI and PAA, demonstrating the viability of this coating strategy with potential biomedical application.

## 5. OAI Evidence from Preclinical and Clinical Studies (Table 2)

Phage therapy has shown promising results for the management of OAIs. A case reported by Fish et al. (2018) documented the clinical resolution of staphylococcal digital osteomyelitis following multiple local phage applications, with successfully preservation of tissue and avoiding the need for amputation [66]. Systematic reviews and larger studies support these findings: Clarke et al. (2020) analyzed 17 reports including 277 patients with bone and joint infections, reporting clinical resolution in 93.1% of cases [67], while Genevière et al. (2021) reported a success rate of 71% in similar cases [17].

In the context of refractory infections, phage therapy has also shown some degree of efficacy. For example, Doub et al. (2023) reported clinical salvage in prosthetic joint infections (PJIs) resistant to antibiotics through local phage application combined with surgical treatment and antibiotics [28]. A larger retrospective study, conducted by Pirnay et al. (2024) and included 100 cases of difficult-to-treat infections, showed clinical improvement in 77.2% of patients and microbiological eradication in 61.3%, highlighting that concomitant use of antibiotics increased treatment efficacy [68].

Recent systematic reviews support these results: Young et al. (2024) estimated a remission rate of 78% in PJIs with various modes of phage application (local, intraoperative, or intraosseous) [22], and Eiselt et al. (2024) concluded that phage therapy is promising as an adjuvant treatment for prosthetic joint infections caused by *S. aureus* and other pathogens, particularly in combination with debridement, antibiotics, and implant retention [69].

Overall, the reviewed studies suggest that phage therapy, whether used as a stand-alone or adjuvant treatment, represents a viable and safe therapeutic strategy for complex bone and joint infections, especially when conventional antibiotics are insufficient.

**Table 2:** Summary of evidence on osteoarticular infections (OAI) from preclinical and clinical studies.

Authors	Study Type/Size	Infection/Intervention	Treatment Regimen (Phages: Single or Alone; Route of Administration; Antibiotics and Outcome)	Ref.
Fish et al., 2018	Case report (compassionate use)	*Staphylococcal* digital osteomyelitis	**Phage:** Single phage: *sb-1* phage specific to *S. aureus.* **Route:** Local injection directly into the affected bone/tissue. **Antibiotics:** Prior levofloxacin therapy failed; phage therapy subsequently added. **Outcome:** Complete clinical resolution; tissue salvaged; amputation avoided.	[66]
Clarke et al., 2020	Systematic review 17 reports, 277 patients	OAI (bone and joint infections)	**Phage:** Both monophage and cocktail regimens were reported across studies (heterogeneous data). **Route:** Predominantly topical/local application; in some cases, included intravenous administration. **Antibiotics:** Most patients received concomitant antibiotic therapy. **Outcome:** Review summarizes overall 93.1% clinical resolution across reports.	[67]
Genevière et al., 2021	Review of bone joint infection cases of 51 patients	Bone and Joint infections	**Phage:** Both monophage and cocktails used. **Route:** Predominantly topical (85% of cases). **Antibiotics**: Used concomitantly in 79% of cases. **Outcome**: Reported overall success rate of 71%.	[17]
Doub et al., 2023	Case report of rescue therapy	Chronic PJI caused by *Enteroccocus faecalis*	**Phage:** Both monophage and cocktails used. **Route:** Intra-articular of the joint. **Antibiotics:** Concomitant antibiotic therapy in addition to phage therapy. **Outcome:** Clinical salvage reported.	[28]
Pirnay et al., 2024	Retrospective multicenter study: 100 cases	Difficult-to-treat infections including bone and OAI	**Phage:** 26 individual phage and 6 predefined cocktails used. **Route:** Varied (local, topical, intravenous). **Antibiotics**: Concomitant use in 69.3% of cases; absence of antibiotics significantly reduced eradication rates. **Outcome**: 77.2% clinical improvement; 61.3% microbiological eradication.	[68]
Young et al., 2024	Systematic review and meta-analysis of 37 patients	PJI	**Phage:** Cocktails used in 65% of cases. **Route:** Mostly intra-articular administration (73% of cases). **Antibiotics:** Combined in 97% of treatments. **Outcome:** Estimated remission rate of 78%.	[22]
Eiselt et al., 2024	Review of 17 publications	PJI caused by *S. aureus*	**Phage:** Both monophage and cocktails discussed. **Route**: Intravenous and intra-articular phage administration. **Antibiotics:** Phage therapy used alongside antibiotics; synergism noted. **Outcome:** Phage therapy reported to be a promising adjuvant	[69]

## 6. OAI Phage Therapy Regulatory Perspective

Phage therapy has not yet been approved for routine use by the EMA or FDA but can be accessed for compassionate use, magistral preparations, or through clinical trials [70,71,72,73,74,75]. Below, we summarize the regulatory and guidance landscape from the FDA relevant to the use of phage therapies in humans, with emphasis on key considerations, pathways, and limitations. Note that while a phage-specific FDA guidance document that fully governs their use has not yet been published, the available materials and statements provide the framework [76].

Key points of the regulatory framework from FDA:

1. Phage therapy is regulated by the FDA Center for Biologics Evaluation and Research (CBER) and phages are classified as biological products (or biologics) when intended for therapeutic use in humans.
2. Because no phage product is currently licensed/approved in the U.S. for human therapeutic use (to date), their use is possible only under investigational pathways (e.g., Investigational New Drug (IND) applications or expanded access).
3. A workshop held by FDA/NIAID (“Science and Regulation of Bacteriophage Therapy”) explored many of the regulatory, manufacturing, quality and trial-design issues for phages.
4. For compassionate/expanded use (single-patient IND, emergency IND, etc.), the FDA expects detailed information on, e.g., phage characterization, manufacture, bacterial host strain matching, sterility, and endotoxins.
5. From a manufacturing/quality perspective: Good Manufacturing Practice (GMP) or an equivalent quality standard is expected, and key aspects such as purity, potency, identity, consistency and sterility must be addressed.
6. Clinical trials of phage therapies must follow similar regulatory principles applied to other biologics: preclinical safety, toxicity, pharmacokinetics/pharmacodynamics, trial protocol, informed consent, and IRB oversight.

The European Medicines Agency (EMA) has recently published documents, i.e., the Concept Paper (2023) and the Draft Guideline on Quality Aspects of Phage Therapy Medicinal Products (2025), which together outline the European regulatory framework in development for bacteriophage-based therapies (EMA/CHMP/BWP/486838/2023) [77] and (EMA/CHMP/BWP/1/2024) [78].

Below we summarize the main aspects of these documents:

Key points of the regulatory framework proposed by the EMA

According to the EMA Concept Paper (EMA/CHMP/BWP/486838/2023) [77], there are two main approaches (or “types”) of phage therapy production and use:

1. Standardized (Predefined) Phage Therapy Medicinal Products (PTMPs):
  - Pre-formulated, fixed-composition medicinal products containing one or more phage strains.
  - The product is manufactured in advance, following GMP and regulatory authorization, similar to other biological medicinal products.
  - Intended for broad or defined bacterial targets (e.g., *S. aureus*, *P. aeruginosa*).
  - Advantages: easy to control, validate, and distribute; suitable for marketing authorization under the standard EU medicinal product framework.
  - Limitation: may lose efficacy if the strain infecting the patient is resistant or not susceptible to phages included.
2. Personalized (Tailored) Phage Therapy Products
  - Active phages are selected or adapted from a pre-existing phage library for bacterial isolate from an individual patient.
  - Production is case-specific, often requiring rapid adaptation or substitution of phages during therapy.
  - This approach allows precision matching between phage and pathogen.
  - Poses major regulatory and manufacturing challenges, including (i) very short timelines for production and testing; (ii) difficulty in maintaining full GMP compliance for each tailored batch, and (iii) complex quality control and documentation requirements.

The regulatory implication of the EMA acknowledges that different quality expectations may be required for these two categories. The forthcoming guidelines mainly focus on standardized PTMPs, although the principles may also apply to personalized use in a risk-adapted manner.

## 7. OAI Phage Therapy Limitations

Despite promising findings, there are some limitations to the current evidence: (i) Most clinical reports are case series; randomized controlled trials are lacking [68]; (ii) long-term outcomes and durability of infection suppression remain unknown; (iii) human pharmacokinetic data in bone and biofilm contexts are scarce [14]; (iv) limited efficacy against intracellular bacteria reduces therapeutic scope (3); (v) phage resistance, stability, and large-scale GMP production pose translational challenges; (vi) synovial inactivation mechanisms and realistic biofilm PD models require deeper characterization [79].

## 8. Conclusions

Phage therapy is a promising approach for managing OAIs, particularly those caused by MDR and biofilm-forming bacteria. Clinical findings, mostly from case and cohort studies, show remission rates of 70–85% in selected cases of PJI treated by a combination of surgery and antibiotics. Although advances in delivery routes and formulations have improved the therapeutic potential, challenges remain regarding GMP-standardized production, pharmacokinetics, safety, and regulation. Future progress will rely on optimized delivery systems, phage–antibiotic combinations, and engineered phages, aiming to establish phage therapy as a standardized, evidence-based treatment for OAIs.

## Figures and Tables

**Table 1:** In vitro and in vivo assays testing different delivery systems for phage therapy in osteoarticular infections.

**In Vitro**					
**Delivery** **System**	**Composition**	**Assay**	**Pathogen**	**Reference**	
Hydrogels	Phage+ Nanohydroxyapatite	Osteoblast Culture	*E. faecalis*	[42]	
	Phage+ Adhesive peptides	Biofilm degradation	*P. aeruginosa*	[43]	
Bone cement	Phage+ Polymethylmethacrylate	Antibacterial activity	*S. aureus*; *P. aeruginosa*	[44]	
Microparticles Nanoparticles	Phage+Silk fibroin+ Polyethylamine	Antibacterial activity	MRSA	[13]	
	Phage+liposome Nanoconjugate	Antibacterial activity; Biofilm degradation	MRSA	[45]	
Implant coating	Phage+hydroxyapatite+ β-TCP	Antibacterial activity	*E. coli*	[46]	
	Phage+hydroxypropyl methylcellulose matrix+linezolid	Antibacterial activity; Bacterial Adhesion	MRSA	[47]	
	Phage+alginate CaCl+ β-TCP	Phage retention	*E. coli*	[48]	
	Phage+polyelectrolytes	Antibacterial activity; Phage adsorption	*E. coli*; *S. aureus*	[49]	
**In Vivo**					
**Delivery System**	**Composition**	**Administration Route**	**Pathogen**	**Model**	**Reference**
Hydrogels	Phage+ Nanohydroxyapatite	Topical	*E. faecalis*	Rabbit	[42]
	Phage + Adhesive peptides	Intra-articular	*P. aeruginosa*	Mouse	[43]
	Phage + DAC^®^ hydrogel	Topical	*S. aureus*	Clinical case	[31]
Bone cement	Phage+ Polymethylmethacrylate	Topical and drainage	MRSA, MRSE, VRE	Clinical case	[10]
Microparticles Nanoparticles	Phage+ Silk fibroin + polyethylamine	Intraperitoneal	MRSA	Mouse	[13]
	Phage + liposome Nanoconjugate	Local injection	MRSA	Rat	[45]
Implant coating	Phage + linezolid in Biopolymer	Implant cover	*S. aureus*	Mouse	[50]

## Data Availability

Data are contained within the article.

## Abbreviations

The following abbreviations are used in this manuscript:

OAIs	Osteoarticular infections
GMP	Good manufacturing production
MRSA	Methicillin-resistant *Staphylococcus Aureus*
PJI	Prosthetic joint infection
DAIR	Debridement, Antibiotics, and Implant Retention
IA	Intra-articular administration
qPCR	Quantitative Polymerase Chain Reaction
CRP	C-Reactive Protein
PMMA	Polymethylmethacrylate
PEMs	Polyelectrolytes
PEI	Polyethyleneimine
FDA	Food, Drug, and Administration
MDR	Multidrug-resistant pathogen
CoNS	Coagulase-negative *Staphylococcus*
Phage	Bacteriophage
PAS	Phage–antibiotic synergy
IV	Intravenous administration
PFU	Plaque forming unit
ESR	Erythrocyte Sedimentation Rate
PAA	Polyacrylic acid
PLGA	Polylactic-co-glycolic
LbL	Layer-by-Layer
EMA	European Medicines Agency
